# A Novel Computational Framework for Predicting the Survival of Cancer Patients With PD-1/PD-L1 Checkpoint Blockade Therapy

**DOI:** 10.3389/fonc.2022.930589

**Published:** 2022-06-27

**Authors:** Xiaofan Su, Haoxuan Jin, Ning Du, Jiaqian Wang, Huiping Lu, Jinyuan Xiao, Xiaoting Li, Jian Yi, Tiantian Gu, Xu Dan, Zhibo Gao, Manxiang Li

**Affiliations:** ^1^ Department of Respiratory and Critical Care Medicine, The First Affiliated Hospital of Xi’an Jiaotong University, Xi’an, China; ^2^ Department of Translational Medicine, YuceBioTechnology Co., Ltd., Shenzhen, China; ^3^ Department of Translational Medicine, YuceNeo Technology Co., Ltd., Shenzhen, China; ^4^ Department of Thoracic Surgery, The First Affiliated Hospital of Xi’an Jiaotong University, Xi’an, China

**Keywords:** gene expression profile, immune exclusion, checkpoint inhibitors, biomarker, prognosis, response

## Abstract

**Background:**

Immune checkpoint inhibitors (ICIs) induce durable responses, but only a minority of patients achieve clinical benefits. The development of gene expression profiling of tumor transcriptomes has enabled identifying prognostic gene expression signatures and patient selection with targeted therapies.

**Methods:**

Immune exclusion score (IES) was built by elastic net-penalized Cox proportional hazards (PHs) model in the discovery cohort and validated *via* four independent cohorts. The survival differences between the two groups were compared using Kaplan-Meier analysis. Both GO and KEGG analyses were performed for functional annotation. CIBERSORTx was also performed to estimate the relative proportion of immune-cell types.

**Results:**

A fifteen-genes immune exclusion score (IES) was developed in the discovery cohort of 65 patients treated with anti-PD-(L)1 therapy. The ROC efficiencies of 1- and 3- year prognosis were 0.842 and 0.82, respectively. Patients with low IES showed a longer PFS (p=0.003) and better response rate (ORR: 43.8% vs 18.2%, p=0.03). We found that patients with low IES enriched with high expression of immune eliminated cell genes, such as CD8+ T cells, CD4+ T cells, NK cells and B cells. IES was positively correlated with other immune exclusion signatures. Furthermore, IES was successfully validated in four independent cohorts (Riaz’s SKCM, Liu’s SKCM, Nathanson’s SKCM and Braun’s ccRCC, n = 367). IES was also negatively correlated with T cell–inflamed signature and independent of TMB.

**Conclusions:**

This novel IES model encompassing immune-related biomarkers might serve as a promising tool for the prognostic prediction of immunotherapy.

## Introduction

Tumor cells acquire numerous genomic alterations, deriving “non-self” neoantigens that the immune system can recognize. Although an immune response is noticed in patients with cancer, this response is usually ineffective to tumor elimination (1–4). One of the reasons is the mechanism of immune escape, including profound local immune suppression, induction of dysfunction, tolerance in T-cell signaling, and evasion of immune destruction by the expression of endogenous “immune checkpoints” that generally lead to immune responses after antigen activation (5). These discoveries have increased cancer understanding and developed immunotherapy treatments such as immune checkpoint inhibitors (ICIs) (6). To date, ICIs therapy like anti-PD-1 has been successful for treating many cancers, particularly malignant melanoma, non-small cell lung cancer, and bladder cancer, among others (7–10).

One challenge of the ICIs immunotherapy is the limited proportion of responders, which leads to the urgent need to find predictive biomarkers to identify responders from non-responders (11). Emerging data suggest that patients overexpressing PD-L1 in tumors by IHC have improved clinical outcomes under anti-PD-1 immunotherapy (12). Although PD-L1 IHC seems predictive in lung cancer, it might not be suitable for many other cancers. The microenvironment of tumors has been recognized as a complex system, as the immune response is affected by many different mechanisms besides PD-L1 (13). Besides, IHC-based detection of PD-L1 as a predictive biomarker is confounded by multiple issues, many still unresolved so far, such as variable detection antibodies and cutoff values and the biomarker’s stability and staining of tumor versus immune cells (12–14).

The development of gene expression profiling within tumors has enabled identifying prognostic gene expression signatures and patient selection (15, 16). Recently reported studies had assessed the association of immune-related gene expression in patients with various solid tumors who received immunotherapy. For instance, a genome-wide analysis of melanoma patients treated with recombinant IL2 revealed a signature predictive of clinical response from pretreatment biopsies (14). Moreover, an IFN-inflammatory immune gene expression signature is associated with both enhanced overall response rates (ORRs) and progression-free survival (PFS) in patients with melanoma who received pembrolizumab, which is subsequently being investigated in other malignancies (17). Other examples include an eight-gene signature reflecting pre-existing immunity, the T-effector/IFN-γ signature, explored in a phase II trial of non–small cell lung carcinoma (NSCLC) (18). Although these studies revealed the intrinsic association between pre-existing immunity and the benefit of ICI therapy, the limitations still exist. On the one hand, these studies failed to consider the functional status of pre-existing immunity, which might affect the outcomes of ICI therapy to a large extent. On the other hand, the selection of those signature genes was mainly based on prior knowledge rather than data exploration, which might lead to insufficient application coverage of these signatures.

Previous research revealed two distinct mechanisms of immune escape in tumor (5; Joyce et al., 2015). One is T cell dysfunction, and the other is T cell exclusion. Approaches that measure immune functional signature based on the gene expression profile were developed to explore the correlation with clinical response of immunotherapy, such as Tumor Immune Dysfunction and Exclusion (TIDE) (19), which identified factors which underlie mechanisms of tumor immune evasion.

So far, most published prognostic gene expression signatures have been explored from the perspective of immune activation and elimination (20, 21). However, as another essential character of the tumor microenvironment, immune-exclusive signatures play a suppression role and are rarely researched. Predicting clinical benefit to ICI therapy requires an understanding of how tumors escape the immune system.

In this study, we evaluated the immune-related gene expression profiles in patients with advanced NSCLC, skin cutaneous melanoma (SKCM), and also head and neck squamous cell carcinoma (HNSCC). We are supposed to find an immune signature that can explain the immune-exclusive statement of tumor samples and can predict response to anti-PD-1 checkpoint inhibitor independently of cancer type.

## Materials and Methods

### Patients and Datasets

This study is a retrospective analysis of patients with immune checkpoint inhibitor therapies. All of the cohorts involved in this study were collected from public datasets, including Prat’s (n=65), Liu’s (n=121), Riaz’s (n=41), Nathanson’s (n=24) and Braun’s (n=181) (22–26). Progression-free survival (PFS) was defined as the beginning of treatment to the date of disease progression (PD). Patients who had not progressed were censored at the date of their last scan. Objective response rate (ORR) was defined as the percentage of patients with complete response (CR) or partial response (PR). Non-objective response (NOR) was defined as the percentage of patients who failed to reach ORR certification. Durable clinical benefit (DCB) was defined as the percentage of patients who achieved CR or PR or stable disease (SD) lasting > 6 months; non-durable clinical benefit (NDB) was defined as PD or SD that lasted ≤ 6 months.

### Immune Exclusion Signature

Firstly, the elastic net-penalized Cox proportional hazards (PHs) model was used to select genes with significant power for predictive value in the discovery cohort. Elastic net, a combination of Ridge and most minor absolute shrinkage and selection operator (LASSO) methods, was applied to select prediction features. The regularization parameter, λ, was specified by 10-fold cross-validation, whereas the L1-L2 trade-off parameter, α, was set to 0.5, with equal Ridge and LASSO penalties. The potential prognostic factors determined by the elastic net-penalized CoxPH regression were subjected to multivariate CoxPH regression analysis to adjust the risk scores of each gene chosen for prognostic clinical parameters. Then, variables exhibiting significance in the adjusted analyzes were entered into a backward, stepwise-elimination Cox regression model. The output calculation formula was: RiskScore = gene expression 1×Coef_1_+gene expression 2×Coef_2_+…+gene expression n×Coef_n_:


$$RiskScore=\sum_{k=1}^{n}(Coef_{k}\times Exp_{k})$$

In this study, the calculation formula of IES was: IES = -2.20*10^-5^*exp(CCL5)-1.55*10^-5^*exp(CCR5)+2.67*10^-7^*exp(CD46)+2.51*10^-5^*exp(CXCL6)+2.97*10^-6^*exp(GPI)-4.40*10^-4^*exp(GZMM)-1.52*10^-3^*exp(IL13)-1.83*10^-3^*exp(IL1RAPL2)+2.10*10^-6^*exp(ITGB1)-1.51*10^-4^*exp(KLRK1)-7.47*10^-5^*exp(NFKB2)-1.66*10^-4^*exp(PDCD1)+1.12*10^-4^*exp(PLA2G6)-2.56*10^-4^*exp(TARP)+5.85*10^-5^*exp(TNFSF4). The software R package cenROC was applied to calculate the ROC of IES for prognostic classification.

### Differentially Expressed Genes

The software R package DESeq2 (V.1.30.1) was used to calculate the fold-change of transcripts and to screen for differentially expressed genes (DEGs) (27) in the RNA-seq data. A fold-change larger than two and an adjusted *p*-value less than 0.05 were set as the cutoff values for screening significant DEGs. Cluster analysis and heatmap generation were performed by the R package and ComplexHeatmap (V.3.12) (25).

### KEGG Pathway, GO and GSEA

All differentially expressed genes were subjected to KEGG term analysis and GO biological processes, including calculation of Benjamini-Hochberg corrected p-values through ToppGene(https://toppgene.cchmc.org/) (28). Gene Set Enrichment Analysis (GSEA) was performed using the GSEA software v.3.0 (Broad Institute, Cambridge, USA) (22).

### Estimation of Immune-Cell Type Fractions

Cell-type identification by estimating relative subsets of RNA transcripts (CIBERSORT), which is a deconvolution algorithm that can characterize the cell proportion of complex tissues, based on LM22, a normalized gene expression profiles (GEPs) (23, 24, 29). In this study, CIBERSORT (https://cibersort.stanford.edu/) and leucocyte signature matrix 22 (LM22) were used to quantify the proportions of immune-cell types HNSCC samples from the TCGA data. Normalized gene expression data were analyzed by the CIBERSORT algorithm, running 1000 permutations. The CIBERSORT p-value reflects the statistical significance of the results, and a threshold less than 0.05 is recommended. Finally, samples with CIBERSORT *p*-values less than 0.05 were included in correlation analyzes between genes and immune-cell types.

### Immune Gene Signatures

Twenty-three independent gene signatures tracking different cell types (e.g., CD8 T cells, NK cells, and Macrophage) and microenvironment (e.g., cytolytic and dysfunction signatures) were evaluated. Correlation coefficients between IES and different gene signatures were calculated. An unsupervised analysis was performed to cluster correlation coefficients with similar values together. The correlation coefficients of each signature can be found in **Supplementary Table S2**.

### Statistical Analysis

Categorical variables were evaluated with Fisher’s exact tests. Correlation analysis was assessed by Pearson coefficient. Multivariable Cox proportional hazards models were built with gene expressions as covariables. Stepwise regression was used to determine the most informative variables included in multiple (linear) regression models. ROC analysis was done using the cenROC package in R. Significance of overall survival (OS) and progression-free survival (PFS) was determined *via* Kaplan-Meier analysis with log-rank analysis. The hazard ratio was calculated by the cox function of the survival package in R. All statistical analysis was performed in the R statistical environment version 3.6.1. All tests were two-tailed and a p-value < 0.05 was considered significant.

## Results

### Immune−Related Gene Expression in the Prognosis of Immunotherapy

To explore the immune-related genes that are related to the prognosis of immunotherapy, we introduced a cohort of 65 patients with advanced NSCLC (n=35), HNSCC (n=5), and SKCM (n=25) from Prat *et al*. (26). Patients were treated with anti-PD-1 monotherapy, and the expression profile of 730 immune-related genes on this cohort was collected. We compared the relationship between clinical characteristics and immune-related gene expression.

**Figure 1:**
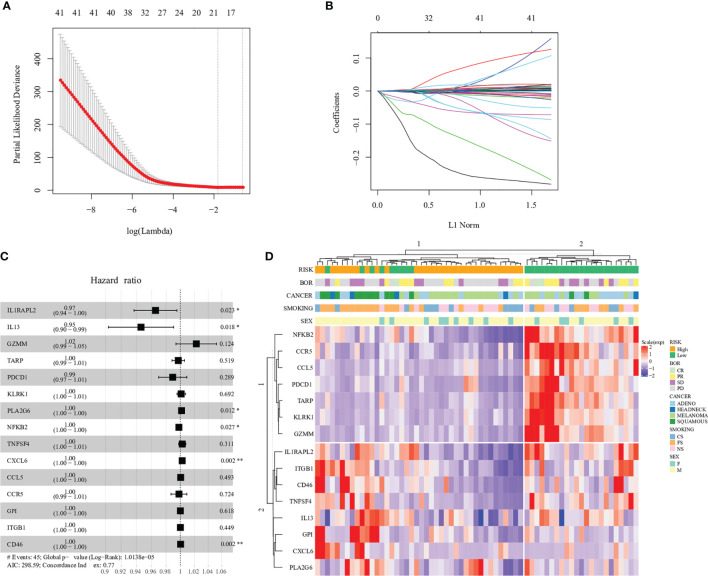
Construction of the immune−related gene model in the prognosis of immunotherapy. **(A)** 1000 bootstrap replicates by lasso Cox regression analysis for variable selection. **(B)** LASSO coefficients of prognosis genes. Each curve represents a prognosis gene. **(C)** Multivariate Cox proportional-hazards model of 15 immune-related genes based on Prat cohort. Forest plot of 15 immune-related genes and their association with clinical survival, with hazard ratio values shown on the y-axis, and p-values derived from multivariate CoxPH analysis. **(D)** Hierarchal clustering analysis of 15 immune-related genes in the Prat cohort. '*',P < 0.05. '**', P < 0.01.

We found that the expression of 41 genes was associated with clinical survival (p < 0.05, respectively) (**Figure S1**). This 41-gene cluster includes complement-related proteins such as C3, C6 and C8A, C-C/C-X-C chemokine ligands/receptors such as CCL5, CCR5, CXCL6 and CXCR3, Interleukin protein families such as IL2 and IL13, Tumor necrosis factor family/superfamily such as TNF and TNFSF4, immune cell surface markers such as CD8A and CD46. The expression of some genes, such as IFNA17 and IL2, had a higher risk associated with prognosis, while others affected patients’ survival slightly. In order to find the gene combinations and their coefficients that are most suitable for prognosis prediction, we applied “Coxnet”, an algorithm that fits cox model regularized by an elastic net penalty (30). Penalty maximum likelihood estimation was performed with 1000 bootstrap replicates (**Figure 1A**). The optimal weighting coefficient of each gene was determined by the regularisation parameter lambda using the 1–SE standard (**Figure 1B**). Overall, fifteen genes, CCL5, CCR5, CD46, CXCL6, GPI, GZMM, IL13, IL1RAPL2, ITGB1, KLRK1, NFKB2, PDCD1, PLA2G6, TARP and TNFSF4, were selected out to explore patient prognosis (**Figure 1C**). The calculation formula of risk score was defined as:

RiskScore = -2.20*10^-5^*exp(CCL5)-1.55*10^-5^*exp(CCR5)+2.67*10^-7^*exp(CD46)+2.51*10^-5^*exp(CXCL6)+2.97*10^-6^*exp(GPI)-4.40*10^-4^*exp(GZMM)-1.52*10^-3^*exp(IL13)-1.83*10^-3^*exp(IL1RAPL2)+2.10*10^-6^*exp(ITGB1)-1.51*10^-4^*exp(KLRK1)-7.47*10^-5^*exp(NFKB2)-1.66*10^-4^*exp(PDCD1)+1.12*10^-4^*exp(PLA2G6)-2.56*10^-4^*exp(TARP)+5.85*10^-5^*exp(TNFSF4)

Among 15 genes, expression of six genes, CD46, CXCL6, GPI, ITGB1, PLA2G6 and TNFSF4, increased the risk of distant recurrence, whereas CCL5, CCR5, GZMM, IL13, IL1RAPL2, KLRK1, NFKB2, PDCD1 and TARP expression had a protective effect against prognosis (**Table S1**). In particular, each single gene expression had little contribution to the higher or lower risk of prognosis, such as PDCD1 (PD-1, hazard ratio: 0.99), which indicated the complexity of the tumor immune microenvironment (**Figure 1C**).

### Prognosis and Clinical Response Prediction With Gene Expression Profile

Fifteen gene expression profiles according to patient prognosis are presented in **Figure 1D**. All patients were divided into two risk groups according to predict scores based on the regression equation of 15 gene expression profiles. The cutoff of low and high risk was based on the median value of the predicted score (cutoff = -0.133). The result of hierarchical cluster analysis was similar to the predicted-score grouping (**Figure 1D**). Patients in the low-risk group were enriched in expression genes of cancer-suppressing inflammation such as NFKB2 and CCR5. In contrast, patients in the high-risk group were enriched in expression genes of cancer-promoting inflammation such as ITGB1 and CD46.

**Figure 2:**
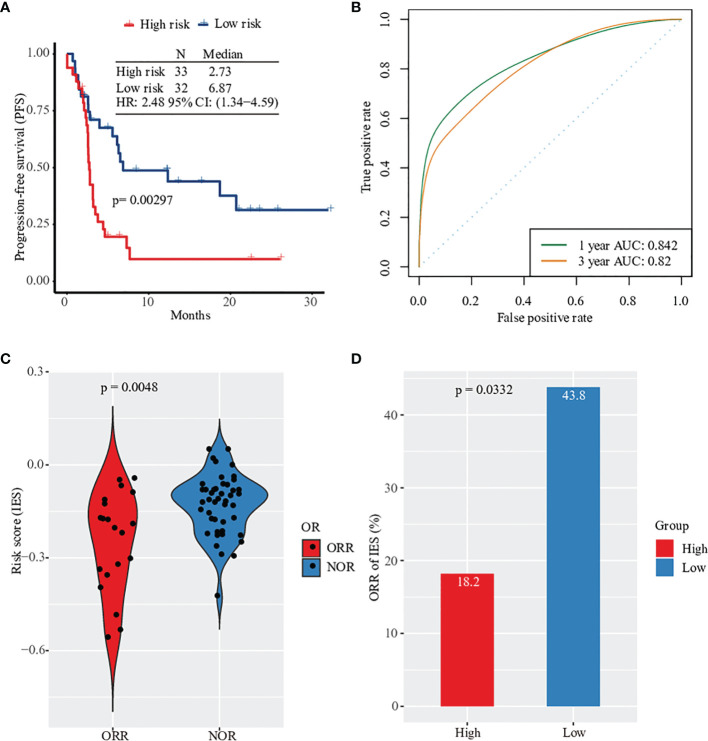
Prognosis and clinical response prediction with gene expression profile. **(A)** Kaplan–Meier survival curves of PFS in high-risk patients versus low-risk patients based on Prat pan-cancer anti-PD-1 monotherapy cohort. **(B)** Sensitivity and specificity of the prognosis risk score model were assessed by time-dependent ROC analysis. **(C)** Violionplot of the distribution of risk score value between patients with ORR and NOR. **(D)** Barplot of object response rate between the high risk group and the low risk group.

To understand the relationship between clinical prognosis and 15 risk genes, we analyzed the correlation between progression-free survival (PFS) and individual gene expression (**Figure S2**). Patients were divided into two groups according to the median expression level. We found that only IL1RAPL2 expression showed a significant correlation in clinical prognosis (**Figure S2**). The expression of the remaining 14 genes tended to predict prognosis to various extents, though it did not reach significance. When merging the expression of 15 genes, patients in the high-risk group had 2.48-fold higher risk of death compared with patients in the low-risk group (hazard ratio [HR]: 2.48, 95% confidence interval [CI]: 1.34, 4.59, p=0.003, **Figure 2A**; **Table S2**). We applied R package cenROC to analysis the ROC efficiencies and found that the area under the ROC curve (AUC) of 1- and 3- year prognosis were 0.842 and 0.82, respectively (**Figure 2B**). Similar results were observed by cancer types of non-squamous NSCLC and SKCM (**Figure S3**). Cancer types of squamous NSCLC and HNSCC did not reach significance due to the small candidate size (**Figure S3**).

Based on the survival analysis results, we evaluated the predicted score and made a prediction model of clinical response in patients. First, we analyzed the predictive performance of every single gene by ROC curve (**Figure S4**). The objective response’s largest AUC was 0.637 of PLA2G6 gene. None of the gene expression was significantly related to the response of anti-PD-1 therapy, including PD-1 (**Figures S5**, **S6**). However, the predictive risk score value from 15 immune-related gene expression levels was observed to increase significantly. This combination predictive model had an AUC of 0.731, higher than the AUC of PD-L1 expression (AUC=0.625, **Figure S7**). Patients without clinical response had a higher value of predicted score (**Figure 2C**). In particular, only 6 of 33 patients in the high-risk group had an objective response after anti-PD-1 therapy (ORR rate: 18.2%) compared with 43.8% of patients in the low-risk group (**Figure 2D**). Together, the expression profile of 15 filtered risk genes was correlated with the prognosis and clinical response of patients treated with anti-PD-1 therapy.

### Pathway and Gene Ontology Analysis Revealed the Difference in Immune Activities Between Two Risk Groups

**Figure 3:**
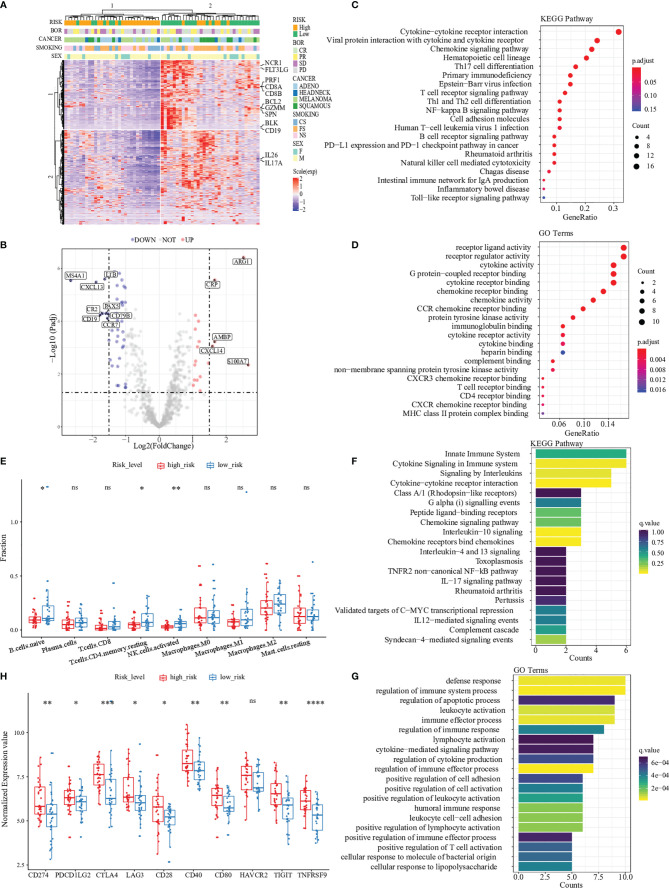
Pathway and gene ontology analysis between two risk groups. **(A)** Hierarchal clustering analysis of 730 immune-related genes, marked with some markers expressed in CD8+ T cells (PRF1, CD8A, CD8B, GZMM and FLT3LG), CD4+ T cells (IL26 and IL17A), NK cells (SPN, BCL2 and NCR1) and B cells (BLK and CD19). **(B)** Differential expression analysis between high-risk patients and low-risk patients in the Prat cohort. “UP” indicates that the gene was significantly up-regulated in the high-risk group while “DOWN” indicates the gene was significantly up-regulated in the low-risk group. **(C, D)** were GO and KEGG enrichment of DEGs, demonstrating that most are related to immune processes. **(E)** The difference of immune cell infiltration abundances between high- and low-risk patients. **(F)** and **(G)** were GO and KEGG enrichment of 15 immune-related genes. **(H)** Different expression in immune checkpoints (CD274, PDCD1LG2, CTLA4, LAG3, CD28, CD40, CD80, HAVCR2, TIGIT, and TNFRSF9) between high- and low-risk patients. '*',P < 0.05.'**',P < 0.01.'***',P < 0.001.’****’,P < 0.0001. “ns”, no significance.

To identify the inner differences in the tumor microenvironment between two risk groups divided by the predicted score of 15 genes expression, an unsupervised analysis of 730 immune-related genes and risk classification was performed in **Figure 3A**. We observed that cluster 2, which mainly consisted of patients with a low-risk score, was enriched with a large number of highly expressed immune eliminated cell genes, such as CD8+ T cells (PRF1, CD8A, CD8B, GZMM and FLT3LG), CD4+ T cells (IL26 and IL17A), NK cells (SPN, BCL2 and NCR1) and B cells (BLK and CD19). Then the analysis of differential expression genes was performed between two risk groups, and the expression of 62 genes (of 730) was identified as statistically altered (p <0.05) (**Figures 3B**, **S8**; **Table S3**). The majority of differentially expressed genes displayed decreased expression in the high-risk group. The greatest downregulation of differential expression gene was MS4A1 (2.64 folds), while the expression of ARG1 and S100A7 in the high-risk group upregulated 2.52 folds and 2.66 folds, respectively.

To better understand the differential expression genes discovered above, we performed pathway enrichment analysis of all differential expression genes between two risk groups by computing their KEGG term and biological process associations. Our analysis generated a total of 19 KEGG terms with a significant p-value (p < 0.05, Benjamini-Hochberg corrected) (**Figure 3C**). Among these KEGG terms, ‘Cytokine-cytokine receptor interaction’ attracted the highest number of differential expression genes, 16 of which seven were discovered among the top twelve differentially expressed transcripts (**Table S4**). After gene ontology analysis, we found ten biological process terms that highly correlated with the differential expression genes (p < 0.001, Benjamini-Hochberg corrected) (**Figure 3D**). Most terms were immune-related receptor activity (i.e., T cell, CCR chemokine and CXCR chemokine) and cytokine activity.

Immune cells are essential components of the tumor microenvironment and closely correlate with immunotherapy responses. We used the CIBERSORT software to assess the abundances of 22 different immune-cell types in 65 patients (**Table S5**). Similar to the findings of KEGG pathways, naïve B cells, CD4 resting-memory T cells, and activated natural killer (NK) cells account for more enormous proportions of the infiltrating immune cells in the low-risk group than in the high-risk group (**Figure 3E**).

### The 15-Gene Expression Profile Is Correlated With Immune-Excluded Microenvironment Characteristics

Path enrichment analysis was also applied to understand the key pathways and biological processes involved in the 15-gene expression profile. Significant pathways and GO terms with two or more enriched genes were selected. The results showed that all of the selected pathways and GO terms were highly relevant to the tumor immune response, such as cytokine signaling, interleukins signaling and lymphocyte activation (**Figures 3F, G**). These critical steps of immune response explained the expression of 15 risk genes that could predictively evaluate the response to immunotherapy.

To further explore the association between the predictive risk score and various immune-related signatures, Pearson correlation analysis was performed to calculate the pairwise correlations among 24 signatures in 65 patients (**Figure S9**). The result of hierarchical clustering revealed that our predictive risk score was positively correlated with M2 macrophage signature, cancer-associated fibroblast (CAF) signature and tumor exclusion signature, while negatively correlated with immune-elimination-related signatures such as cytotoxic T lymphocytes (CTL) signature, MHC-II signature and interferon-gamma (IFN-γ) signature. This observation helps us understand that patients with high predicted risk scores received worse clinical outcomes could be explained by the immune exclusion status in their tumor microenvironment to some extends. We also found that the predictive risk score was positively correlated with the expression of classical immune checkpoints, such as PD1, PD-L1, and CTLA4 (**Figure 3H**). Thus, we termed our 15-gene risk score as “Immune Exclusion Score” (IES).

### Validation of IES Score in Multiple ICIs Therapy Cohorts

**Figure 4:**
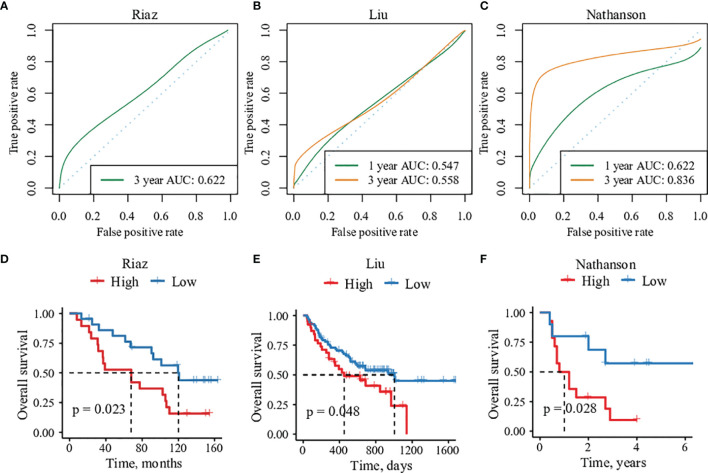
The predictive efficacy of 15 immune-related genes risk score in three validation cohorts. **(A–C)** Sensitivity and specificity of the risk score model were assessed in each dataset by time-dependent ROC analysis. **(D–F)** Overall survival analysis between high- and low-risk groups in each cohorts.

To validate the robustness and eligibility of the IES, we collected three more cohorts that underwent ICIs treatments. All of the patients in the three cohorts were with advanced melanoma, including Liu cohort (n=121), Riaz cohort (n=41) and Nathanson cohort (n=24) (31–33). IES scores were calculated among patients in three cohorts and applied to predict the clinical response of immunotherapy (**Tables S6**–**S8**). ROC analysis showed that the ROC efficiencies of 1- prognosis were 0.547 and 0.622 in Liu and Nathanson cohort, respectively (1- year prognosis AUC was unevaluable in Riaz cohort). While the ROC efficiencies of 3- year prognosis were 0.622, 0.558 and 0.836 in Riaz, Liu and Nathanson cohort, respectively (**Figures 4A–C**). Patients were divided into IES high and low groups according to the Youden index and the threshold value were -0.0006, -0.0001 and -0.0015 in Riaz, Liu and Nathanson cohort, respectively. We observed a better survival rate and more extended survival advantage in patients within the IES low group in all of the three cohorts (**Figures 4D–F**). In the Riaz and Nathanson cohorts, the cutoff value was close to the median value of IES among patients. We found that patients with high IES were correlated with worse clinical response in three cohorts, while the objective response rates of the low IES group among three cohorts were 31.8%, 43.9% and 60%, respectively (**Figure S10**). These results confirmed the predictive performance of clinical outcomes of IES in immunotherapy.

Since the cancer type of all three cohorts used for verification above was melanoma, we introduced another immunotherapy cohort of clear-cell renal cell carcinoma(ccRCC) from Braun et al. (34). The ccRCC cohort contains 181 patients treated with anti-PD-1 therapy. Previously studies revealed that traditional biomarkers of immunotherapy such as PD-L1 and TMB didn’t show the ability to distinguish the clinical outcome in ccRCC and the microenvironment of CD8+ T-cell infiltration was related to poor prognosis of immunotherapy. After calculating the IES of ccRCC patients, survival analysis and ROC analysis were applied to evaluate the performance of IES. Intriguingly, our results indicated that patients with high IES, whose tumor microenvironment had the feature of immune exclusion, were correlated with longer PFS (p=0.02, IES cutoff=-0.0976) (**Figure S11**; **Table S9**). This result was consistent with the findings in ccRCC, although different from the majority of understanding in the tumor microenvironment. Previous studies proved that *PBRM1* mutation was promoting factor of ccRCC ICIs therapy. Thus, we applied IES in the *PBRM1*-mut subgroup of the ccRCC cohort (**Figure S11**). The findings in the datasets above showed that patients with high IES scores showed a worse prognosis of OS in the *PBRM1*-mut subgroup, which indicated that IES could further filter patients with worse clinical outcomes and prognosis ICIs therapy who acquired *PBRM1* mutations.

### Comparison Between IES and Other Biomarkers of Immunotherapy

Recently, the expression of genes related to cytolytic immune activity was associated with clinical response to ICIs in certain tumors (35, 36). A previous study discovered a T cell–inflamed 18-gene expression profile (GEP) shown to predict response to anti–PD-1 therapy (37). To compare the performance on clinical response to anti-PD-1 therapy between GEP and IES, T cell–inflamed GEP was assessed in all patients from the Prat cohort, Liu cohort, Riaz cohort and Nathanson cohort (**Figure S12**). We found that higher T cell–inflamed GEP scores were also positively associated with response and prognosis in Prat cohort, Liu cohort, Riaz cohort and Nathanson cohort, showing that the T cell–activated tumor environment also affects response in addition to IES. However, significance was not demonstrated in the subgroups by cancer types in Prat cohort when using GEP as a predictor. We also found that a higher proportion of responders were enriched in the “low-risk” group by IES, compared with GEP.

**Figure 5:**
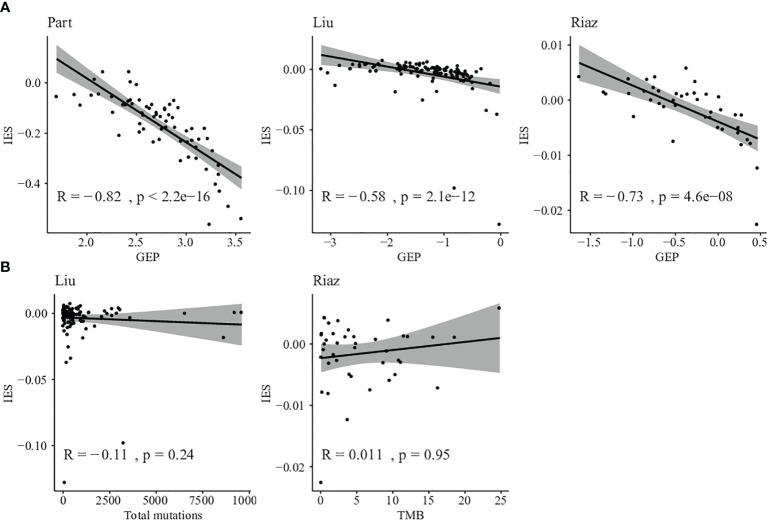
Comparison between IES and other biomarkers. **(A)** Correlation between T cell–inflamed GEP score and IES score in Prat, Liu, and Riaz cohorts. **(B)** Correlation between somatic mutation counts or TMB and IES score in Liu and Riaz cohorts.

We next evaluated the correlation between GEP and IES among those patients. Negative correlations were found between GEP and IES, and 57.1% of shared patients were selected as low risk by both GEP and IES (**Figure 5A**). So IES could be a necessary complement to GEP.

Furthermore, we evaluated the relationship between TMB and IES in Liu and Riaz cohort. IES score was found independent of TMB in the discovery and validation datasets with correlations of -0.11 and 0.011, respectively (**Figure 5B**). This result indicated that the IES score could be applied independently or jointly with TMB in predicting the response of ICIs therapy.

## Discussion

The immune checkpoint inhibitors (ICIs) have made remarkable progress in the clinical treatment of tumors in the past decade (38–40). However, immune escape mechanisms and immune resistance to ICIs therapy have not been well-studied. Here, we discovered and developed an immune exclusion-related 15-gene risk score termed “Immune Exclusion Score” (IES) to predict the clinical response and prognosis to anti-PD-1 therapy. Limited clinical outcomes and prognosis to anti-PD-1 treatment occurred in patients with high IES risk scores in the discovery and validation datasets. Besides, IES was also found as a necessary complement to T cell-inflamed GEP signature and independent to TMB, reflective of the relationship of GEP and TMB to IES. These observations suggest that using the IES immune exclusion biomarker may help identify patients who are responsive to anti–PD-1 therapies and explain the mechanism of immune exclusion in the tumor microenvironment.

In this study, we focused on 15 immune-related gene expressions as a prognostic marker of ICIs therapy. Among these genes, CCL5, CCR5 and PDCD1 are essential regulators of T-cell antigen receptor signaling (41–43). Gene GZMM, KLRK1 and NFKB2 activate and improve the cytolytic activity of T and NK cells (44–46). While the high expression of gene CD46, CXCL6 and GPI inhibit the inflammation of the tumor microenvironment (47–49). Our results revealed that CCL5, CCR5, GZMM, IL13, IL1RAPL2, KLRK1, NFKB2, PDCD1 and TARP expression were strong protective effects against distant recurrence of ICIs. The rest genes, CD46, CXCL6, GPI, ITGB1, PLA2G6 and TNFSF4, were revealed for the negative association with tumor suppression.

The differential gene expression and pathway analysis were evaluated between two risk groups divided by the IES scores. Our results showed that patients with low IES score enriched with highly expressed immune eliminated cell genes of CD8+ T cells, CD4+ T cells, NK cells and B cells. Most of the enriched pathways were related to immune-cell membrane receptor activity and cytokine activity. We also observed that naïve B cells, CD4 resting-memory T cells, and activated natural killer (NK) cells infiltrated more enormous proportions in the low IES group than in the high IES group.

Our study results may explain the low response rates and the limited efficacy of ICIs for patients with high IES scores. The IES was positively correlated with immune exclusion signatures such as M2 macrophage signature and CAF signature. In contrast, IES was negatively correlated with immune-elimination-related signatures such as CTL signature, MHC-II signature and IFN-γ signature. In addition, the IES score was positively correlated with the high expression of immune checkpoints like PD1, PD-L1 and CTLA4, which indicated the status of immune suppression among IES-high patients.

The limitations of this study still exist. Firstly, the development and validation of IES were conducted on four retrospective cohorts. Prospective clinical studies are needed to verify the clinical efficacy of IES as a predictive biomarker for immunotherapy. Secondly, the prognostic model is built based only on gene expression data. A model involving more types of data, especially pathological images might be able to improve the prediction accuracy (50, 51). Thirdly, due to the limited size of patients/cohorts, IES was confirmed in a small number of cancer types, such as NSCLC, melanoma, ccRCC and NPC. Data on more cancer types are needed to prove the broad applicability of IES. Besides, all of the cohorts introduced in this study were treated with ICI monotherapy. Combined treatment approaches with ICIs, such as ICI combined with chemotherapy, demonstrated a superior clinical response in recent trials. A more significant implication will illustrate if IES is successfully validated in the combination of ICIs and chemotherapy.

## Conclusions

Our data demonstrate that IES can be used to categorize tumors into different subgroups that exhibit distinct patterns of potentially recognizable biology to enhance clinical response. Although the utility of IES, T cell–inflamed GEP and TMB, as well as other emerging agnostic biomarkers, need further validated for response prediction to various immunotherapy approaches, including combination therapies, these findings provide the possibility for further exploring the utility of these biomarkers as guides for clinical precision cancer immunotherapy.

## Data Availability Statement

The original contributions presented in the study are included in the article/**Supplementary Material**. Further inquiries can be directed to the corresponding author.

## Author Contributions

XS, HJ, ZG, and ML contributed to research design. Data analysis was carried out by HJ, JW, JX, and XL. JY and XD developed the algorithm and refined the prediction model. ND, HL, and TG collected clinical information that was used in the study and critically revised the manuscript. XS, HJ, and ND wrote the manuscript. All authors provided feedback and approved the final version.

## Conflict of Interest

Authors XS, HJ, JW, HL, JX, XL, JY, TG, XD, and ZG were employed by YuceBioTechnology Co., Ltd., Shenzhen, China.

The remaining authors declare that the research was conducted in the absence of any commercial or financial relationships that could be construed as a potential conflict of interest

## Publisher’s Note

All claims expressed in this article are solely those of the authors and do not necessarily represent those of their affiliated organizations, or those of the publisher, the editors and the reviewers. Any product that may be evaluated in this article, or claim that may be made by its manufacturer, is not guaranteed or endorsed by the publisher.

## Supplementary Material

Supplementary Figure 1 Forest plot of 41 immune-related genes and their association with clinical survival.

Supplementary Figure 2 Kaplan–Meier survival curves of PFS in high-expression patients versus low-expression patients grouped by 15 immune-related genes median expression level in Prat cohort.

Supplementary Figure 3 Kaplan–Meier survival curves of PFS in high-risk patients versus low-risk patients with advanced SKCM (n=25), LUAD (n=22), HNSCC (n=5), and LUSC (13) in Prat cohort.

Supplementary Figure 4 The ROC curves of the IES score and 15 immune-related genes expression level for predicting response in the Prat cohort.

Supplementary Figure 5 Objective response rate (ORR) between high- and low-expressed patients of 15 immune-related genes in patients in the Prat cohorts. ORR is defined as having a partial response (PR) or a complete response (CR); NOR is defined as having no PR or CR.

Supplementary Figure 6 The different expression level of 15 immune-related genes between ORR and NOR patients in the Prat cohort. ORR is defined as having a partial response (PR) or a complete response (CR); NOR is defined as having no PR or CR. ‘ns’, not significant. ‘*’, P < 0.05.

Supplementary Figure 7 The ROC curves and AUC values of the risk score from 15 immune-related genes expression level and PD-L1 expression level for predicting objective response in the Prat cohort. ‘ns’, not significant.

Supplementary Figure 8 Hierarchal clustering analysis of DEGs, split by the high- and low-risk groups.

Supplementary Figure 9 Pairwise correlation coefficients heatmap among 24 signatures in **(A)** Prat cohort, **(B)** Liu cohort and **(C)** Riaz cohort.

Supplementary Figure 10 Prediction efficacy of IES for clinical response in three validation datasets. **(A)** The ROC curves and AUC values in each cohort. **(B)** Objective response rate (ORR) between high- and low-risk patients in the three cohorts. ORR is defined as having a partial response (PR) or a complete response (CR); NOR is defined as having no PR or CR.

Supplementary Figure 11 The predictive value of IES score in ccRCC cohort. **(A)** ROC curves and AUC values, **(B)** Objective response rate (ORR), and **(C)** Kaplan–Meier survival curves of PFS between high- and low-risk patients in the total ccRCC cohort, PBRM1-mut subset, and PBRM1-wt subset. ORR is defined as having a partial response (PR) or a complete response (CR); NOR is defined as having no PR or CR.

Supplementary Figure 12 The predictive value of GEP score in Prat, Liu, Riaz, and Nathanson cohorts. **(A)** Objective response rate (ORR) and **(B)** Kaplan–Meier survival curves of PFS or OS between high- and low-risk patients in the four cohorts. ORR is defined as having a partial response (PR) or a complete response (CR); NOR is defined as having no PR or CR.

## Abbreviations

IES, immune exclusion score; ICIs, immune checkpoint inhibitors; GEPs, gene expression profiles; NK, activated natural killer; CAF, cancer-associated fibroblast; TCGA, The Cancer Genome Atlas; GO, Gene Ontology; KEGG, Kyoto Encyclopedia of Genes and Genomes; GSEA, Gene Set Enrichment Analysis; NSCLC, non-small cell lung cancer; SKCM, skin cutaneous melanoma; HNSCC, head and neck squamous cell carcinoma; ccRCC, clear-cell renal cell carcinoma; NPC, nasopharyngeal carcinoma; anti-PD-(L)1, anti-programmed death-(ligand) 1; MHC, major histocompatibility complex; ORR, objective response rate; NOR, non-objective response; DCB, durable clinical benefit; NDB, non-durable clinical benefit; CR, complete response; PR, partial response; SD, stable disease; PD, disease progression; PFS, progression-free survival; OS, overall survival; TMB, tumor mutation burden; ROC, receiver operating characteristic curve; AUC, area under ROC curve; HIC, immunohistochemistry.

## References

[1] Mellman I Coukos G Dranoff G. Cancer Immunotherapy Comes of Age. Nature (2011) 480(7378):480–9. doi: 10.1038/nature10673 PMC3967235 22193102

[2] Topalian SL Weiner GJ Pardoll DM. Cancer Immunotherapy Comes of Age. J Clin Oncol (2011) 29(36):4828–36. doi: 10.1200/jco.2011.38.0899 PMC3255990 22042955

[3] Zhang W Yin Z Sun Z Tian Y Wang Y. Selecting Transferrable Neurophysiological Features for Inter-Individual Emotion Recognition *via* a Shared-Subspace Feature Elimination Approach. Comput Biol Med (2020) 123:103875. doi: 10.1016/j.compbiomed.2020.103875 32658790

[4] Alar HS Fernandez PL. Accurate and Efficient Mosquito Genus Classification Algorithm Using Candidate-Elimination and Nearest Centroid on Extracted Features of Wingbeat Acoustic Properties. Comput Biol Med (2021) 139:104973. doi: 10.1016/j.compbiomed.2021.104973 34717231

[5] Gajewski TF Schreiber H Fu YX. Innate and Adaptive Immune Cells in the Tumor Microenvironment. Nat Immunol (2013) 14(10):1014–22. doi: 10.1038/ni.2703 PMC4118725 24048123

[6] Topalian SL Hodi FS Brahmer JR Gettinger SN Smith DC McDermott DF. Safety, Activity, and Immune Correlates of Anti-PD-1 Antibody in Cancer. N Engl J Med (2012) 366(26):2443–54. doi: 10.1056/NEJMoa1200690 PMC3544539 22658127

[7] Brahmer JR Tykodi SS Chow LQ Hwu WJ Topalian SL Hwu P. Safety and Activity of Anti-PD-L1 Antibody in Patients With Advanced Cancer. N Engl J Med (2012) 366(26):2455–65. doi: 10.1056/NEJMoa1200694 PMC3563263 22658128

[8] Garon EB Rizvi NA Hui R Leighl N Balmanoukian AS Eder JP. Pembrolizumab for the Treatment of non-Small-Cell Lung Cancer. N Engl J Med (2015) 372(21):2018–28. doi: 10.1056/NEJMoa1501824 25891174

[9] Robert C Schachter J Long GV Arance A Grob JJ Mortier L. Pembrolizumab Versus Ipilimumab in Advanced Melanoma. N Engl J Med (2015) 372(26):2521–32. doi: 10.1056/NEJMoa1503093 25891173

[10] Bellmunt J de Wit R Vaughn DJ Fradet Y Lee JL Fong L. Pembrolizumab as Second-Line Therapy for Advanced Urothelial Carcinoma. N Engl J Med (2017) 376(11):1015–26. doi: 10.1056/NEJMoa1613683 PMC5635424 28212060

[11] Yarchoan M Hopkins A Jaffee EM. Tumor Mutational Burden and Response Rate to PD-1 Inhibition. N Engl J Med (2017) 377(25):2500–1. doi: 10.1056/NEJMc1713444 PMC6549688 29262275

[12] Patel SP Kurzrock R. PD-L1 Expression as a Predictive Biomarker in Cancer Immunotherapy. Mol Cancer Ther (2015) 14(4):847–56. doi: 10.1158/1535-7163.Mct-14-0983 25695955

[13] Balkwill FR Capasso M Hagemann T. The Tumor Microenvironment at a Glance. J Cell Sci (2012) 125(Pt 23):5591–6. doi: 10.1242/jcs.116392 23420197

[14] Weiss GR Grosh WW Chianese-Bullock KA Zhao Y Liu H Slingluff CL Jr. Molecular Insights on the Peripheral and Intratumoral Effects of Systemic High-Dose rIL-2 (Aldesleukin) Administration for the Treatment of Metastatic Melanoma. Clin Cancer Res (2011) 17(23):7440–50. doi: 10.1158/1078-0432.Ccr-11-1650 PMC3229653 21976537

[15] Liu H Qiu C Wang B Bing P Tian G Zhang X. Evaluating DNA Methylation, Gene Expression, Somatic Mutation, and Their Combinations in Inferring Tumor Tissue-Of-Origin. Front Cell Dev Biol (2021) 9:619330. doi: 10.3389/fcell.2021.619330 34012960 PMC8126648

[16] Yang M Yang H Ji L Hu X Tian G Wang B. A Multi-Omics Machine Learning Framework in Predicting the Survival of Colorectal Cancer Patients. Comput Biol Med (2022) 146:105516. doi: 10.1016/j.compbiomed.2022.105516 35468406

[17] Ribas A Robert C Hodi FS Wolchok JD Joshua AM Hwu W-J. Association of Response to Programmed Death Receptor 1 (PD-1) Blockade With Pembrolizumab (MK-3475) With an Interferon-Inflammatory Immune Gene Signature. J Clin Oncol (2015) 33(15_suppl):3001–1. doi: 10.1200/jco.2015.33.15_suppl.3001

[18] Fehrenbacher L Spira A Ballinger M Kowanetz M Vansteenkiste J Mazieres J. Atezolizumab Versus Docetaxel for Patients With Previously Treated non-Small-Cell Lung Cancer (POPLAR): A Multicentre, Open-Label, Phase 2 Randomised Controlled Trial. Lancet (2016) 387(10030):1837–46. doi: 10.1016/s0140-6736(16)00587-0 26970723

[19] Jiang P Gu S Pan D Fu J Sahu A Hu X. Signatures of T Cell Dysfunction and Exclusion Predict Cancer Immunotherapy Response. Nat Med (2018) 24(10):1550–8. doi: 10.1038/s41591-018-0136-1 PMC6487502 30127393

[20] Nema R Shrivastava A Kumar A. Prognostic Role of Lipid Phosphate Phosphatases in non-Smoker, Lung Adenocarcinoma Patients. Comput Biol Med (2021) 129:104141. doi: 10.1016/j.compbiomed.2020.104141 33260104

[21] Tu M Ye L Hu S Wang W Zhu P Lu X. Identification of Glioma Specific Genes as Diagnostic and Prognostic Markers for Glioma. Curr Bioinf (2021) 16(1):120–9. doi: 10.2174/1574893615999200424090954

[22] Subramanian A Tamayo P Mootha VK Mukherjee S Ebert BL Gillette MA. Gene Set Enrichment Analysis: A Knowledge-Based Approach for Interpreting Genome-Wide Expression Profiles. Proc Natl Acad Sci U S A (2005) 102(43):15545–50. doi: 10.1073/pnas.0506580102 PMC1239896 16199517

[23] Yoshihara K Shahmoradgoli M Martínez E Vegesna R Kim H Torres-Garcia W. Inferring Tumour Purity and Stromal and Immune Cell Admixture From Expression Data. Nat Commun (2013) 4:2612. doi: 10.1038/ncomms3612 24113773 PMC3826632

[24] Newman AM Liu CL Green MR Gentles AJ Feng W Xu Y. Robust Enumeration of Cell Subsets From Tissue Expression Profiles. Nat Methods (2015) 12(5):453–7. doi: 10.1038/nmeth.3337 PMC4739640 25822800

[25] Ding Y Tang J Guo F. Identification of Protein–Protein Interactions *via* a Novel Matrix-Based Sequence Representation Model With Amino Acid Contact Information. Int J Mol Sci (2016) 17(10):1623. doi: 10.3390/ijms17101623 PMC5085656 27669239

[26] Prat A Navarro A Paré L Reguart N Galván P Pascual T. Immune-Related Gene Expression Profiling After PD-1 Blockade in Non-Small Cell Lung Carcinoma, Head and Neck Squamous Cell Carcinoma, and Melanoma. Cancer Res (2017) 77(13):3540–50. doi: 10.1158/0008-5472.Can-16-3556 28487385

[27] Love MI Huber W Anders S. Moderated Estimation of Fold Change and Dispersion for RNA-Seq Data With Deseq2. Genome Biol (2014) 15(12):550. doi: 10.1186/s13059-014-0550-8 25516281 PMC4302049

[28] Chen J Bardes EE Aronow BJ Jegga AG. ToppGene Suite for Gene List Enrichment Analysis and Candidate Gene Prioritization. Nucleic Acids Res (2009) 37(Web Server issue):W305–311. doi: 10.1093/nar/gkp427 PMC2703978 19465376

[29] Chen B Khodadoust MS Liu CL Newman AM Alizadeh AA. Profiling Tumor Infiltrating Immune Cells With CIBERSORT. Methods Mol Biol (2018) 1711:243–59. doi: 10.1007/978-1-4939-7493-1_12 PMC5895181 29344893

[30] Friedman J Hastie T Tibshirani R. Regularization Paths for Generalized Linear Models *via* Coordinate Descent. J Stat Softw (2010) 33(1):1–22. doi: 10.18637/jss.v033.i01 20808728 PMC2929880

[31] Nathanson T Ahuja A Rubinsteyn A Aksoy BA Hellmann MD Miao D. Somatic Mutations and Neoepitope Homology in Melanomas Treated With CTLA-4 Blockade. Cancer Immunol Res (2017) 5(1):84–91. doi: 10.1158/2326-6066.Cir-16-0019 27956380 PMC5253347

[32] Riaz N Havel JJ Makarov V Desrichard A Urba WJ Sims JS. Tumor and Microenvironment Evolution During Immunotherapy With Nivolumab. Cell (2017) 171(4):934–49.e916. doi: 10.1016/j.cell.2017.09.028 29033130 PMC5685550

[33] Liu D Schilling B Liu D Sucker A Livingstone E Jerby-Arnon L. Integrative Molecular and Clinical Modeling of Clinical Outcomes to PD1 Blockade in Patients With Metastatic Melanoma. Nat Med (2019) 25(12):1916–27. doi: 10.1038/s41591-019-0654-5 PMC6898788 31792460

[34] Braun DA Hou Y Bakouny Z Ficial M Sant' Angelo M Forman J. Interplay of Somatic Alterations and Immune Infiltration Modulates Response to PD-1 Blockade in Advanced Clear Cell Renal Cell Carcinoma. Nat Med (2020) 26(6):909–18. doi: 10.1038/s41591-020-0839-y PMC7499153 32472114

[35] Tumeh PC Harview CL Yearley JH Shintaku IP Taylor EJ Robert L. PD-1 Blockade Induces Responses by Inhibiting Adaptive Immune Resistance. Nature (2014) 515(7528):568–71. doi: 10.1038/nature13954 PMC4246418 25428505

[36] Rooney MS Shukla SA Wu CJ Getz G Hacohen N. Molecular and Genetic Properties of Tumors Associated With Local Immune Cytolytic Activity. Cell (2015) 160(1-2):48–61. doi: 10.1016/j.cell.2014.12.033 25594174 PMC4856474

[37] Ayers M Lunceford J Nebozhyn M Murphy E Loboda A Kaufman DR. IFN-γ-Related mRNA Profile Predicts Clinical Response to PD-1 Blockade. J Clin Invest (2017) 127(8):2930–40. doi: 10.1172/jci91190 PMC5531419 28650338

[38] Darvin P Toor SM Sasidharan Nair V Elkord E. Immune Checkpoint Inhibitors: Recent Progress and Potential Biomarkers. Exp Mol Med (2018) 50(12):1–11. doi: 10.1038/s12276-018-0191-1 PMC6292890 30546008

[39] Wilky BA. Immune Checkpoint Inhibitors: The Linchpins of Modern Immunotherapy. Immunol Rev (2019) 290(1):6–23. doi: 10.1111/imr.12766 31355494

[40] Ramos-Casals M Brahmer JR Callahan MK Flores-Chávez A Keegan N Khamashta MA. Immune-Related Adverse Events of Checkpoint Inhibitors. Nat Rev Dis Primers (2020) 6(1):1–21. doi: 10.1038/s41572-020-0160-6 32382051 PMC9728094

[41] Lapteva N Huang XF. CCL5 as an Adjuvant for Cancer Immunotherapy. Expert Opin Biol Ther (2010) 10(5):725–33. doi: 10.1517/14712591003657128 20233026

[42] Ribas A Wolchok JD. Cancer Immunotherapy Using Checkpoint Blockade. Sci (New York N.Y.) (2018) 359(6382):1350–5. doi: 10.1126/science.aar4060 PMC7391259 29567705

[43] Jiao X Nawab O Patel T Kossenkov AV Halama N Jaeger D. Recent Advances Targeting CCR5 for Cancer and Its Role in Immuno-Oncology. Cancer Res (2019) 79(19):4801–7. doi: 10.1158/0008-5472.Can-19-1167 PMC6810651 31292161

[44] Ishimaru N Kishimoto H Hayashi Y Sprent J. Regulation of Naive T Cell Function by the NF-Kappab2 Pathway. Nat Immunol (2006) 7(7):763–72. doi: 10.1038/ni1351 16732290

[45] Roufas C Chasiotis D Makris A Efstathiades C Dimopoulos C Zaravinos A. The Expression and Prognostic Impact of Immune Cytolytic Activity-Related Markers in Human Malignancies: A Comprehensive Meta-Analysis. Front Oncol (2018) 8:27. doi: 10.3389/fonc.2018.00027 29515971 PMC5826382

[46] Lazarova M Steinle A. The NKG2D Axis: An Emerging Target in Cancer Immunotherapy. Expert Opin Ther Targets (2019) 23(4):281–94. doi: 10.1080/14728222.2019.1580693 30732494

[47] Tsai YG Lai JC Yang KD Hung CH Yeh YJ Lin CY. Enhanced CD46-Induced Regulatory T Cells Suppress Allergic Inflammation After Dermatophagoides Pteronyssinus-Specific Immunotherapy. J Allergy Clin Immunol (2014) 134(5):1206–09.e1201. doi: 10.1016/j.jaci.2014.06.005 25065720

[48] Hussein NH Amin NS El Tayebi HM. GPI-AP: Unraveling a New Class of Malignancy Mediators and Potential Immunotherapy Targets. Front Oncol (2020) 10:537311. doi: 10.3389/fonc.2020.537311 33344222 PMC7746843

[49] Zheng S Shen T Liu Q Liu T Tuerxun A Zhang Q. CXCL6 Fuels the Growth and Metastases of Esophageal Squamous Cell Carcinoma Cells Both *In Vitro* and *In Vivo* Through Upregulation of PD-L1 *via* Activation of STAT3 Pathway. J Cell Physiol (2021) 236(7):5373–86. doi: 10.1002/jcp.30236 33368292

[50] Yang J Ju J Guo L Ji B Shi S Yang Z. Prediction of HER2-Positive Breast Cancer Recurrence and Metastasis Risk From Histopathological Images and Clinical Information *via* Multimodal Deep Learning. Comput Struct Biotechnol J (2022) 20:333–42. doi: 10.1016/j.csbj.2021.12.028 PMC8733169 35035786

[51] Ye Z Zhang Y Liang Y Lang J Zhang X Zang G. Cervical Cancer Metastasis and Recurrence Risk Prediction Based on Deep Convolutional Neural Network. Curr Bioinf (2022) 17(2):164–73. doi: 10.2174/1574893616666210708143556

