# Supplementary figures and images for: A Novel Computational Framework for Predicting the Survival of Cancer Patients With PD-1/PD-L1 Checkpoint Blockade Therapy
Source: Front Oncol. 2022 Jun 27;12:930589. doi: 10.3389/fonc.2022.930589 (PMC9271954; doi:10.3389/fonc.2022.930589)

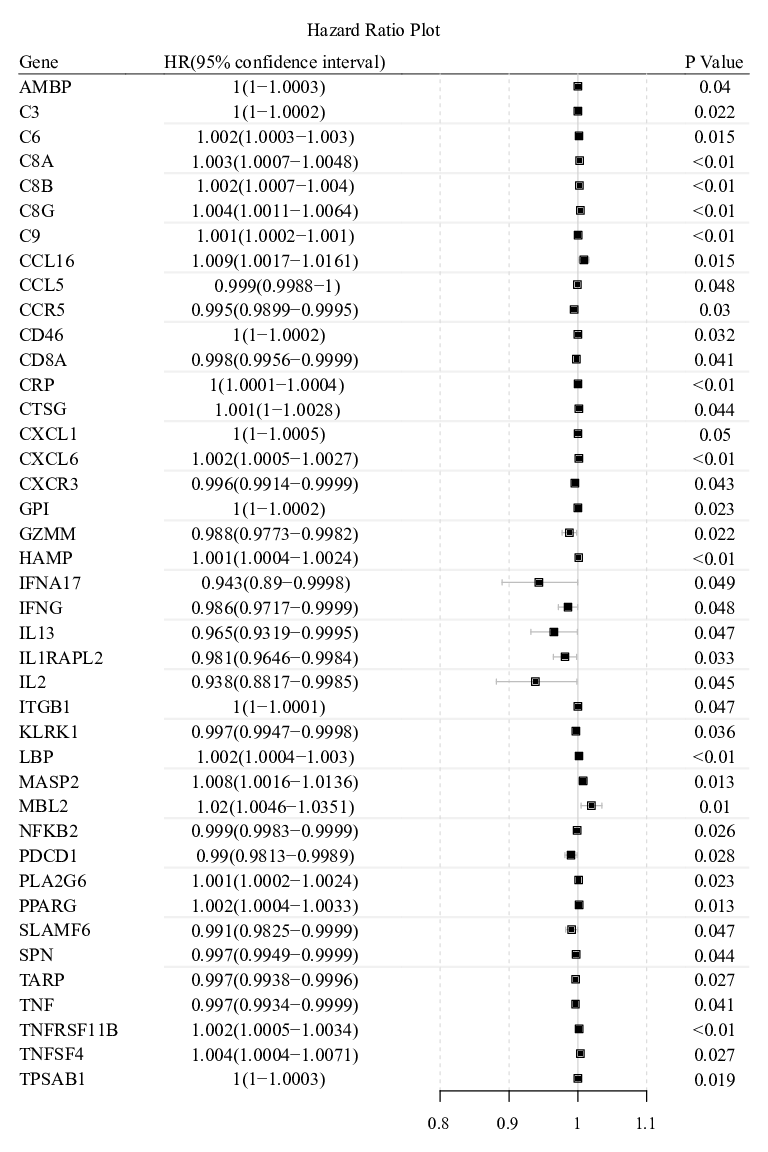

Supplement: Supplementary Figure 1 — Forest plot of 41 immune-related genes and their association with clinical survival. [file DataSheet_1.zip › Supplementary Materials/FigureS1.tiff]

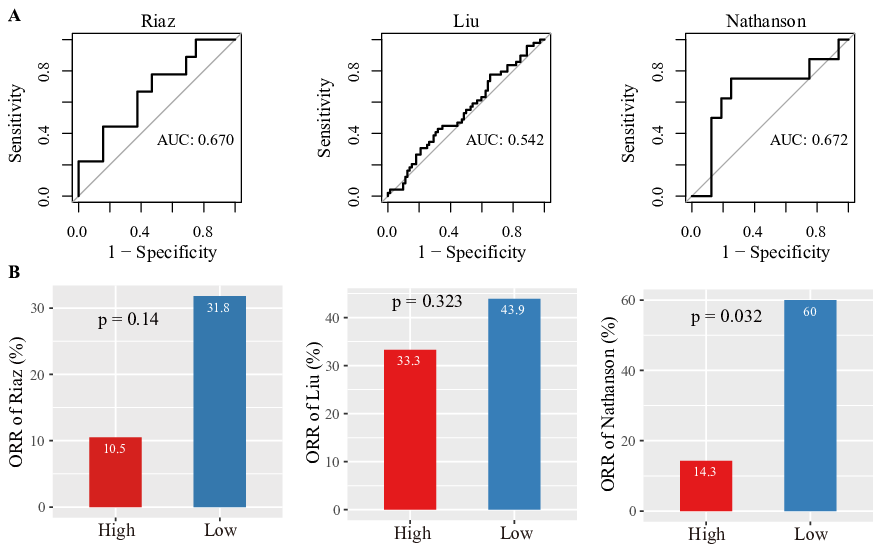

Supplement: Supplementary Figure 1 — Forest plot of 41 immune-related genes and their association with clinical survival. [file DataSheet_1.zip › Supplementary Materials/FigureS10.tiff]

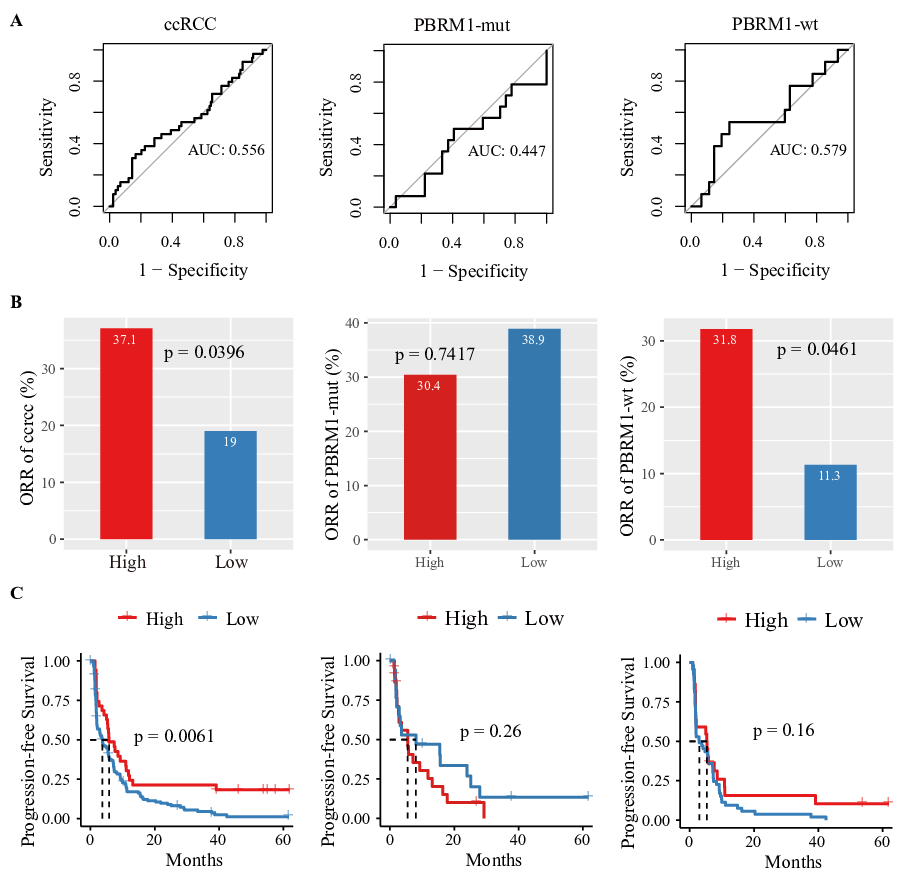

Supplement: Supplementary Figure 1 — Forest plot of 41 immune-related genes and their association with clinical survival. [file DataSheet_1.zip › Supplementary Materials/FigureS11.tiff]

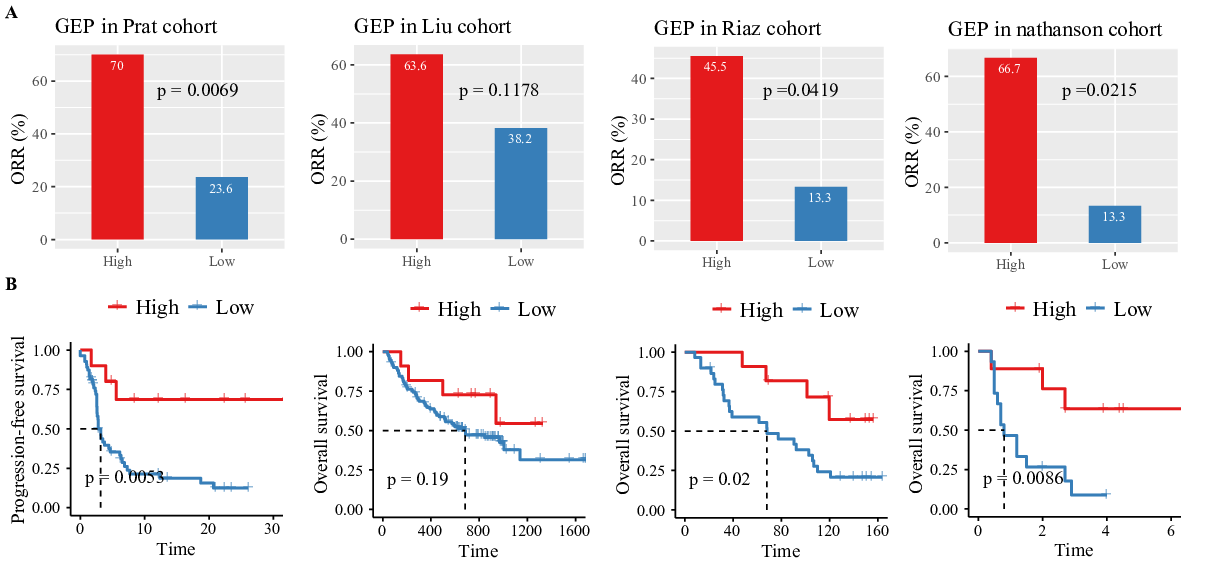

Supplement: Supplementary Figure 1 — Forest plot of 41 immune-related genes and their association with clinical survival. [file DataSheet_1.zip › Supplementary Materials/FigureS12.tiff]

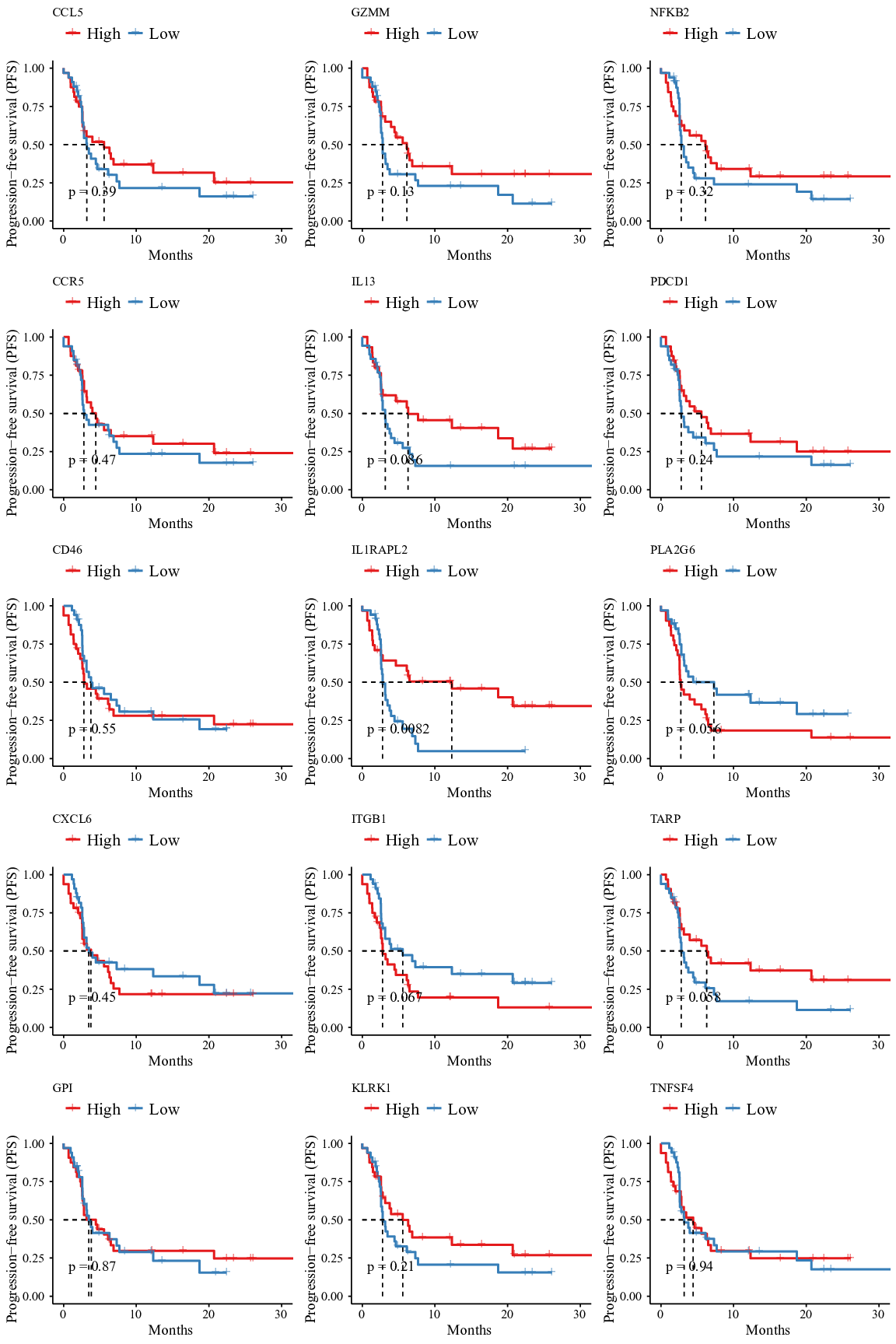

Supplement: Supplementary Figure 1 — Forest plot of 41 immune-related genes and their association with clinical survival. [file DataSheet_1.zip › Supplementary Materials/FigureS2.tiff]

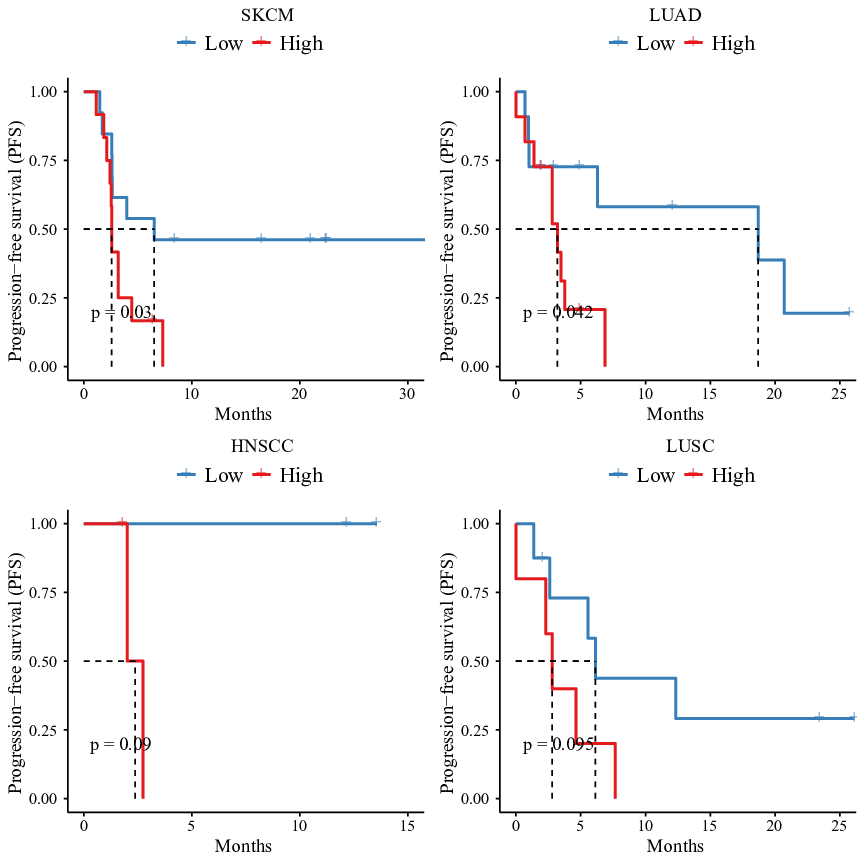

Supplement: Supplementary Figure 1 — Forest plot of 41 immune-related genes and their association with clinical survival. [file DataSheet_1.zip › Supplementary Materials/FigureS3.tiff]

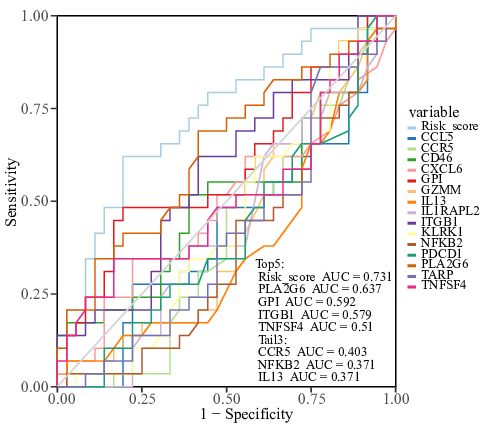

Supplement: Supplementary Figure 1 — Forest plot of 41 immune-related genes and their association with clinical survival. [file DataSheet_1.zip › Supplementary Materials/FigureS4.tiff]

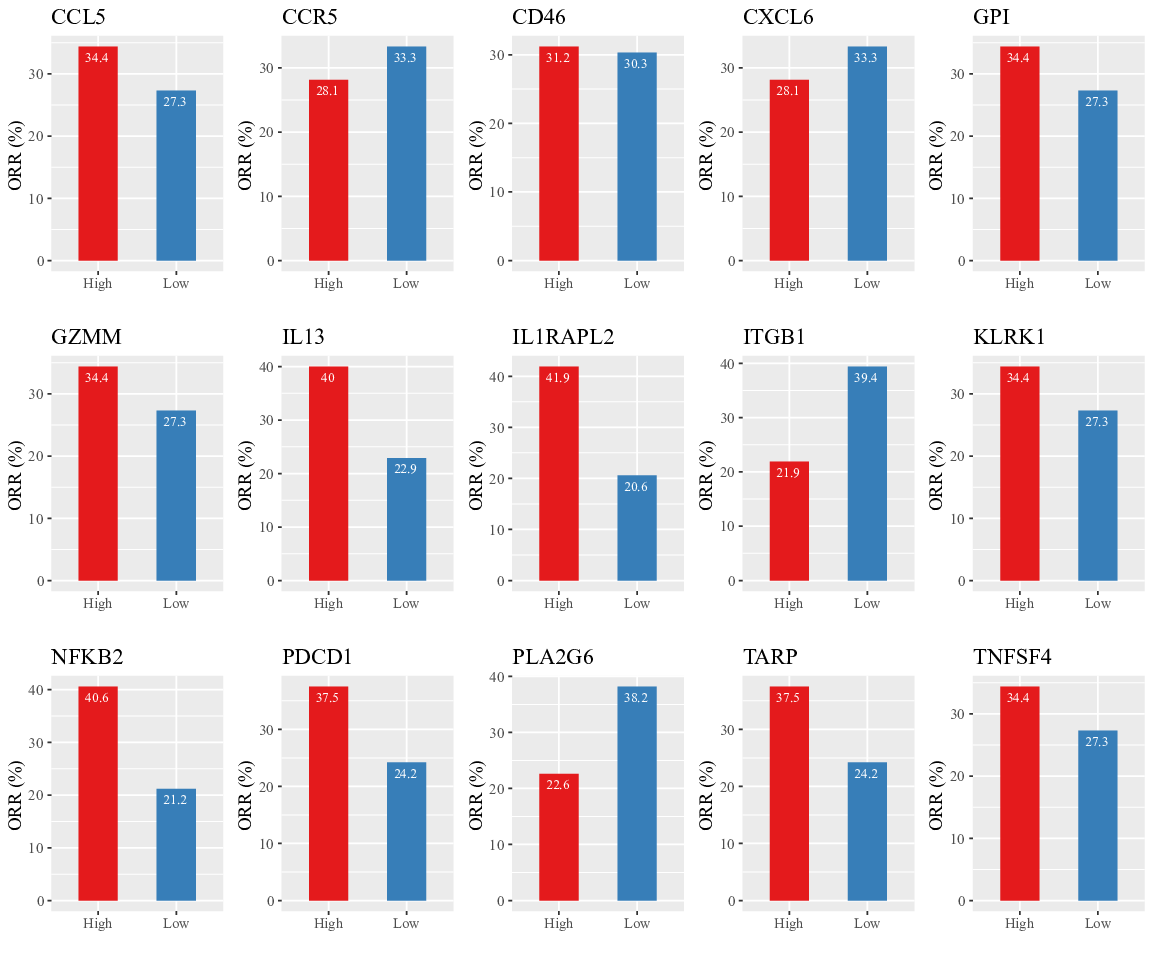

Supplement: Supplementary Figure 1 — Forest plot of 41 immune-related genes and their association with clinical survival. [file DataSheet_1.zip › Supplementary Materials/FigureS5.tiff]

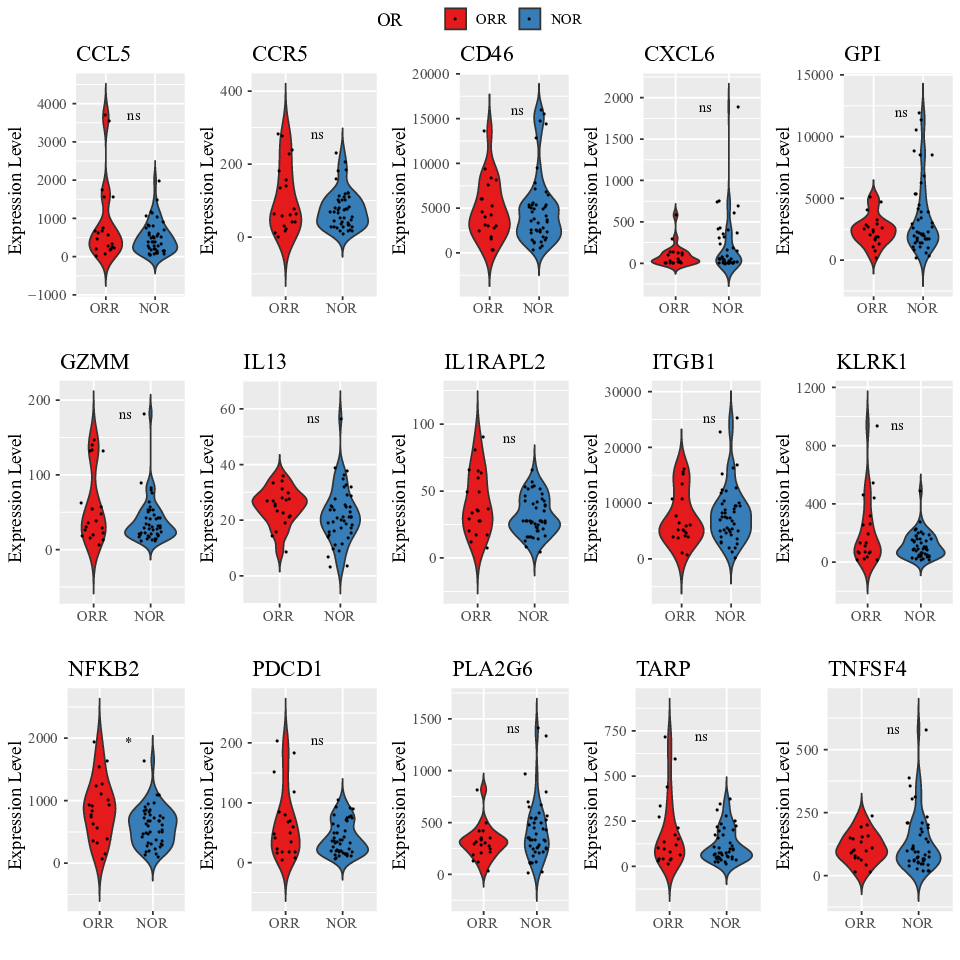

Supplement: Supplementary Figure 1 — Forest plot of 41 immune-related genes and their association with clinical survival. [file DataSheet_1.zip › Supplementary Materials/FigureS6.tiff]

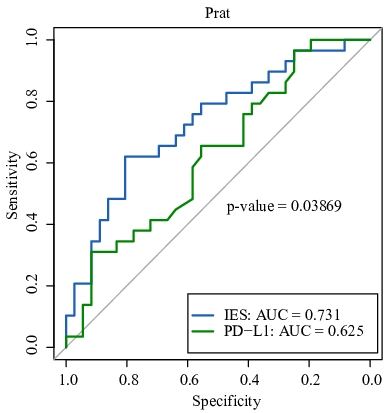

Supplement: Supplementary Figure 1 — Forest plot of 41 immune-related genes and their association with clinical survival. [file DataSheet_1.zip › Supplementary Materials/FigureS7.tiff]

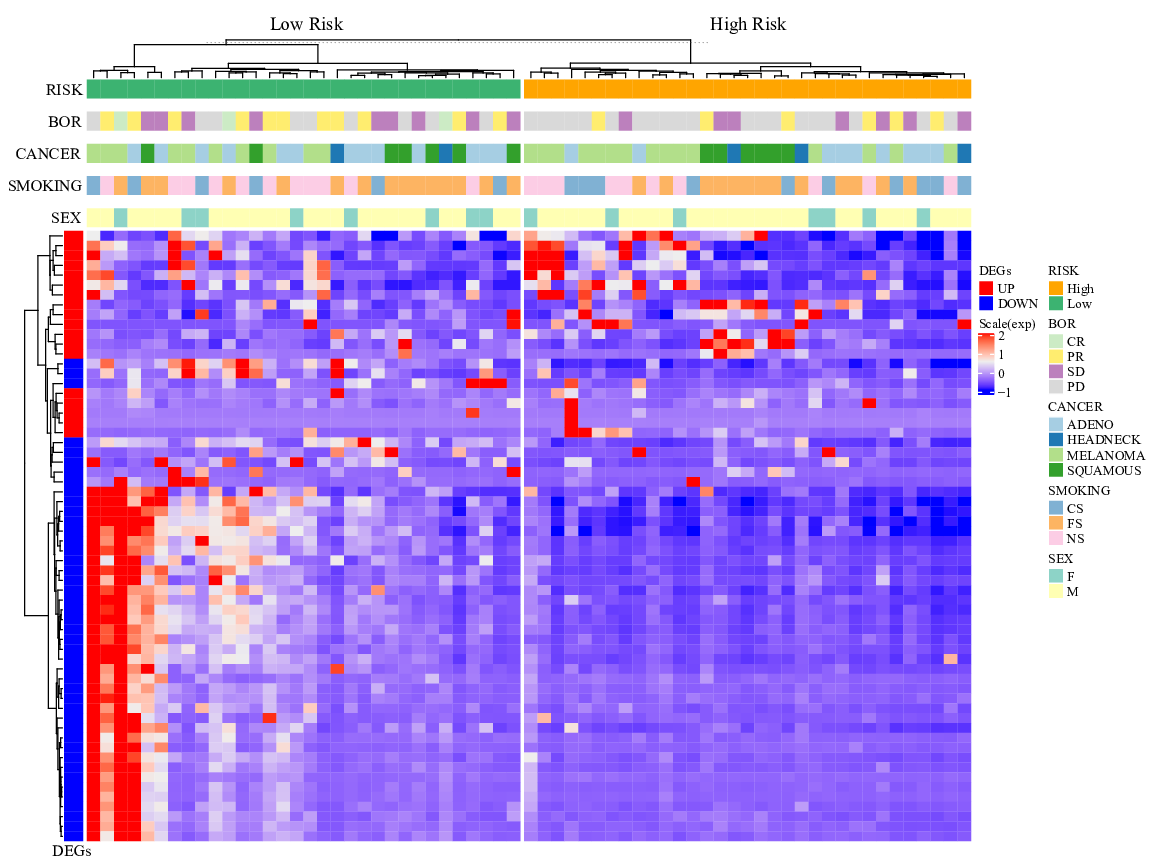

Supplement: Supplementary Figure 1 — Forest plot of 41 immune-related genes and their association with clinical survival. [file DataSheet_1.zip › Supplementary Materials/FigureS8.tiff]

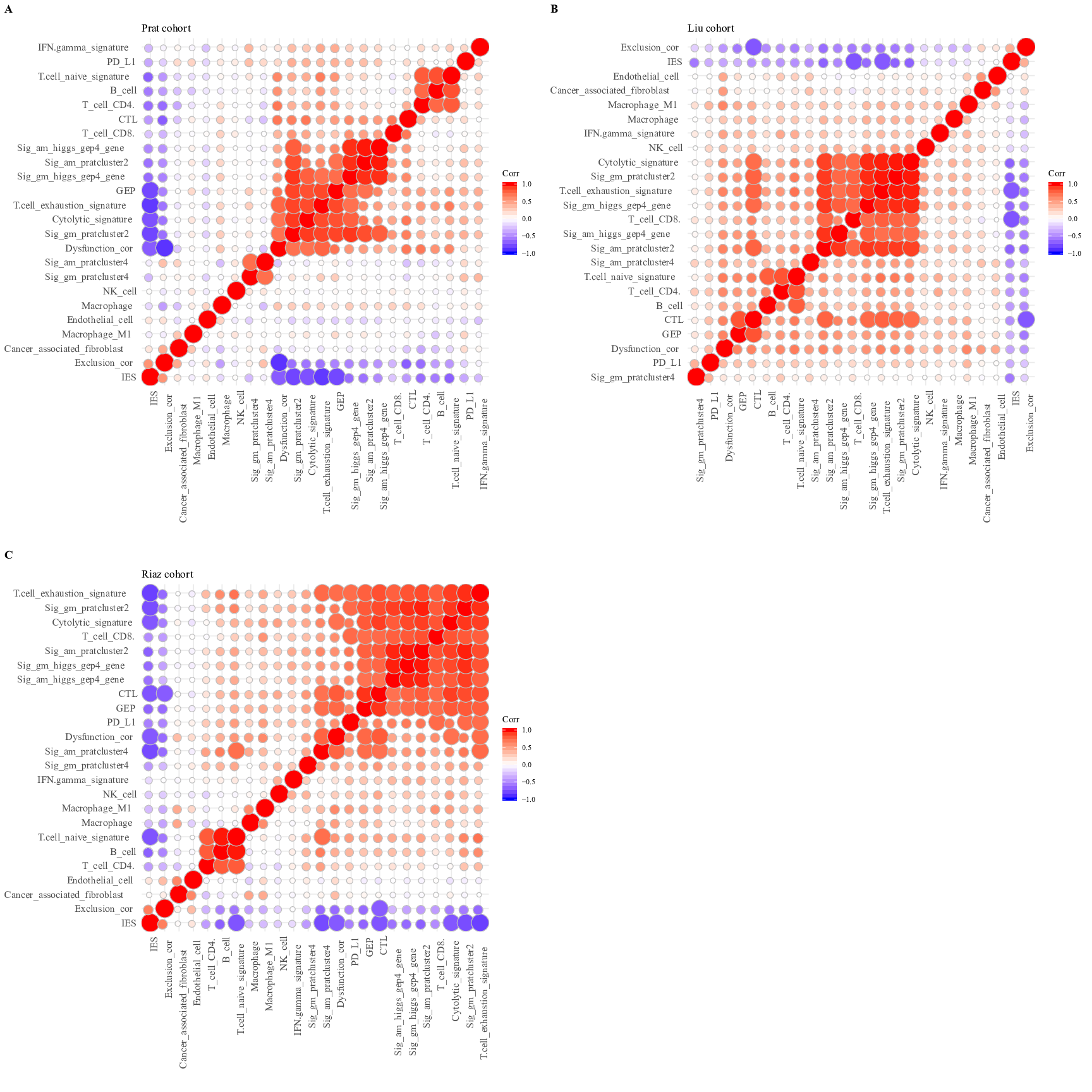

Supplement: Supplementary Figure 1 — Forest plot of 41 immune-related genes and their association with clinical survival. [file DataSheet_1.zip › Supplementary Materials/FigureS9.tiff]
